# Synthesis, characterization, and evaluation of KDM4B inhibitors to attenuate inflammatory host immune response in periodontitis

**DOI:** 10.1007/s00044-024-03362-5

**Published:** 2024-12-13

**Authors:** Kathleen A. Garrabrant, Amelia B. Furbish, Jonathan M. Turner, Ivett Pina Gomez, Catherine M. Mills, Abhiram Maddi, Yuri K. Peterson

**Affiliations:** 1 Department of Drug Discovery and Biomedical Sciences, Medical University of South Carolina, Charleston, SC, USA; 2 Department of Medicine, Massachusetts General Hospital, Boston, MA, USA; 3 Department of Pathology and Laboratory Medicine, Charleston, SC, USA; 4 Department of Advance Specialty Sciences, Medical University of South Carolina, Charleston, SC, USA

**Keywords:** Periodontal disease, KDM4B, epigenetics, inflammation, medicinal chemistry

## Abstract

Periodontal disease begins with bacterial plaque buildup in the oral cavity, inciting an inflammatory response that results in subsequent tissue damage. Even after standard treatment like scaling and root planning (SRP) to remove plaque and biofilm, the host immune response can remain hyper-active, perpetuating further tissue destruction. In these cases, aggressive periodontitis is resistant to SRP and the inflammatory response may persist, even in the absence of plaque, presenting a significant clinical challenge. Previous experiments have provided a validated model of periodontal inflammation by exposing murine macrophages to lipopolysaccharide (LPS) from *Aggregatibacter actinomycetemcomitans* (*Aa*), a pathogen linked to aggressive periodontitis. Using this model, we have previously demonstrated that the periodontal disease microenvironment triggers epigenetic changes, notably heightened lysine-specific demethylase 4B (KDM4B) activity. Data indicate that the KDM4B inhibitor ML324 can reverse the macrophage-mediated pro-inflammatory response induced by *Aa* LPS in vitro, providing compelling evidence for KDM4B as a rational therapeutic target for periodontal disease. In the present studies, a cohort of compounds was developed as potential KDM4B inhibitors. Synthesis and characterization of derivatives led to the discovery of compound **14** with an IC_50_ of 170 nM against KDM4B and immunosuppressive activity in the *Aa* LPS challenge model. These results suggest KDM4B inhibitors as potential therapeutic agents for modulating the immune response for periodontal disease.

## Graphical Abstract

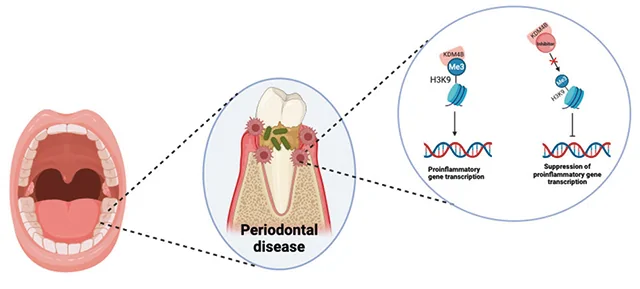

## Introduction

Periodontal disease (PD) is a condition associated with dysbiosis of the oral microbiome in a vulnerable host, affecting almost 50% of the adult population in the United States and approximately 743 million people worldwide [1]. In addition to the prevalence of PD also represents a significant economic burden, contributing to substantial healthcare costs and productivity losses worldwide [2, 3]. The global impact of PD underscores the need for effective and sustainable treatment strategies to target the various aspects of disease progression.

The mildest form of PD, gingivitis, is described as the inflammation of the gingiva due to the accumulation of bacteria around the gum line of the tooth. Unlike PD, gingivitis is reversible upon improvement of oral hygiene. Once gingival inflammation has progressed passed the stage of gingivitis, it is classified as PD, an irreversible inflammatory disease state associated with significant tissue destruction. In the setting of PD, microorganisms can penetrate the gingival tissues, triggering an inflammatory immune response that results in further destruction of the periodontium, attachment loss, and alveolar bone loss [4]. The oral bacteria responsible for the initiation and propagation of disease include several key pathogens, notably *Aggregatibacter actinomycetemcomitans* and *Porphyromonas gingivalis* [4]. Although the presence of periopathogenic bacteria is essential for disease pathology, it is the host immune response to microbial challenge that leads to immune cell-mediated tissue damage and, in severe cases, bone loss [5].

The current standard of care for PD consists of scaling and root planing (SRP) supplemented with home care review [6]. Current nonsurgical treatments focus on the removal of dental biofilm as well the addition of antimicrobial therapies, such as antibiotics or antiseptics in conjunction with SRP. Although antibiotics/antiseptics are generally effective for short-term treatment, SPR and adjunctive antimicrobial therapies are insufficient for longterm management due to antibiotic resistance and the persistence of subclinical inflammation [7]. Recently, the use of minocycline microspheres was found to be a safe and efficient adjunctive therapy to the current standard of care and produced a significant reduction in clinical parameters of PD [8]. Despite these new advances, disease recurrence is common, driven by continued immune cell activation and impaired periodontal wound healing [9, 10]. As such, there is a significant clinical need for the development of novel treatment strategies that directly target immune cells and modulate the destructive host inflammatory response, allowing host immune cells to resolve infection and subsequent periodontal wound healing to occur. Adjunctive therapies may be able to halt PD progression and have potential utility in patients who progress to develop a hyperinflammatory response against the microbiome, and for cases where conventional therapies prove ineffective.

PD has been associated with many co-morbidities, including rheumatoid arthritis, obesity, cardiovascular disease, colorectal cancer, and oral squamous cell carcinoma [11, 12]. Studies have shown that inflammation originating in the oral cavity can exacerbate systemic inflammatory responses, highlighting the broader health implications of PD. Currently, there are several host immune-modulating agents being studied in periodontitis across a number of cellular targets such as osteoblast/osteoclast activity, NF-κB, adenosine, TNF-α, and more [5, 13–15], many of which represent emerging drug candidates in clinical trials [5]. Preclinical studies have shown the success of shifting from inhibiting individual inflammatory mediators themselves to converting the inflammatory condition into a noninflammatory one, effectively resolving the local and systemic inflammation associated with PD [16]. These findings highlight the need to complement current treatment options with strategies that adjunctively target immune cells to modulate host inflammatory response, allowing wounds to heal, and preventing disease reoccurrence, however, there are currently no approved therapeutics available for this use.

Gingival tissue in patients with PD is known to have higher expression of proinflammatory cytokines (PIC) that promote tissue destruction. This process is thought to be stimulated by periodontopathogens through TLR4 signaling in M1 macrophages [17]. In addition, a number of cytokines have been implicated in PD pathogenesis including IL-6, which promotes subsequent B-cell activation and IL-1 production, and TNF-α, which shows synergistic effects with IL-1 and promotes bone resorption [18]. Epigenetic modifications, such as those mediated by lysine-specific demethylases, play crucial roles in regulating gene expression and immune responses [19]. Recent advancements in epigenetic research has opened new avenues for therapeutic intervention in chronic inflammatory diseases [20]. The gene transcription of PICs is regulated, in part, by these epigenetic enzymes [21], including the ‘erasers’ of the lysine-specific demethylase 4 (KDM4) family, which act on specific methylated lysine residues, serving as ‘switches’ to regulate gene transcription. One particular homolog of this family, KDM4B, and its substrate, histone 3 lysine 9 (H3K9), have been implicated in immune activation and PIC release [22, 23]. Tri-methylated histone 3 lysine 9 (H3K9me3) is shown to be a repressive methyl marker, and removal of this marker results in increased inflammatory gene expression [24]. KDM4B acts to demethylate in a 2-step oxidated decarboxylation reaction, using Fe(II), 2-oxoglutarate, and oxygen as cofactors [25]. KDM4B is a key regulator of inflammatory responses and bone resorption, and targeting this enzyme offers a promising therapeutic approach [26, 27].

KDM4B has been implicated as a regulatory enzyme in periodontal disease progression through its role in suppressing both osteoclastogenesis and inflammatory cytokine production in macrophages [28]. Additionally, KDM4B inhibition by the compound ML324 results in suppression of PIC production as well as osteoclast formation in murine macrophages. While ML324 exhibits good cellular membrane permeability, it is highly insoluble at effective doses and requires a 14-step synthesis for derivatization [29]. The binding pocket of Jumonji domain-containing histone demethylases poses significant challenges for small molecule drug discovery. While a number of carboxylic acid-containing compounds have been identified, they show limited or no activity in cells. Strategies such as carboxylic acid bioisosteric substituent and pro-drug formulation have been attempted [30, 31]. Although developing such compounds is difficult, drugs with carboxylic acid moieties are among the most successful in the pharmaceutical market [32]. Currently, 2-OG binding site inhibitors are being developed for the prolyl-hydroxylase-domain containing protein (PHD2). An ideal approach for future development of KDM4 inhibitors would be to balance the polarity of carboxylic acids with lipophilicity and c of heteroatoms could produce inhibitors with favorable drug-like properties. While small molecule KDM4B inhibitors **1-4** (Fig. 1) have been developed, they also present significant therapeutic challenges [29, 33–35]. These challenges include poor selectivity and specificity over other JmjC domain demethylases, low potency, and inadequate cell penetration. We sought to identify novel, synthetically accessible scaffolds with the ability to suppress PIC release through KDM4B inhibition and potentially serve as optimizable compounds for localized treatment of periodontal disease.

This study aimed to explore novel KDM4B inhibitors as a potential therapeutic strategy for PD, through modulation of the epigenetic mechanisms underlying inflammation and tissue destruction to prevent disease progression. In this work, we describe a multidisciplinary approach to the identification, synthesis, and characterization of a library of novel benzamidobenzoic acid derivatives demonstrating potent KDM4B inhibitory activity and phenotypic immunosuppression. We further used this strategy to optimize a number of lead compounds demonstrating therapeutic potential for the treatment of periodontal disease based on suppression of the immune response through an epigenetic mechanism

## Results and Discussion

### Chemical Synthesis

We first tested a biphenyl benzamide scaffold with a carboxamide linker (Table 1). Compound **6** showed acceptable inhibitory activity but had poor synthetic efficiency. Further in silico studies were coupled with the enzymatic inhibitory screen to move into a second group of compounds featuring a 2-aminobenzoic acid system, as well as to the biphenyl system at the active 4-position. This generation of compounds yielded increased inhibitory activity and improved the synthesis. From this the compounds expanded to investigate changes within the linker type and functional groups. Functional groups explored include various halogens and methylsimilar moieties (i.e., methyl, methoxy, trifluoromethyl). Further modification of the biphenyl system to various [4.4.0] bicyclo systems (*i.e.*, naphthyl, tetrahydro naphthyl, and benzodioxanyl) and saturated cyclohexyl benzyl was performed as well, with compound **6** (Scheme 1) being used as a reference for further studies to compare efficacy activity. We also explored bioisosteric substitution of the carboxylic acid through synthesis of tetrazoles using copper-catalyzed cycloaddition of azide with nitriles and of the carboxamide through synthesis of sulfonamides from sulfonyl chlorides (Scheme 2) as previously described [36]. Commercially available aryl sulfonyl chlorides were reacted with the appropriately substituted methy 2-aminobenzoates via nucleophilic acyl substitution, followed by methyl ester cleavage to yield 2-(arylsulfonamido) benzoic acids. In a similar manner, reaction of the aryl sulfonyl chlorides with substituted 2-aminobenzonitiriles yielded the corresponding coupled products. This was followed by [2 + 3] cycloaddition of the azide to the nitrile to produce the N-[2-(1H-tetrazol-5yl) phenyl]benzenesulfonamide. The group of compounds that were explored for activity against KDM4B, shown in Table 1, were selected to explore structure activity relationship (SAR) to improve compound potency.

### KDM4B inhibitory activity and IC50 analysis

Derivatives were tested for enzyme inhibition using a primary screen for inhibitory activity against KDM4B at 20 μM (Fig. 2), where a bulk histone assay was employed to determine methylation response as previously described [37]. The compounds that displayed 50% ≥ inhibition were selected for IC_50_ determination. The core structures are designated as Stx 1 and Stx 2, with the latter being more prevalent among the series, displaying varied activities depending on the different substituents R1-R5. Substituents at R1 predominately feature carboxylic acid, which is known to be favorable for activity in KDM4B inhibitor [32]. Compound **8**, with a tetrazole moiety at R1, shows promising potency (IC_50_ of 0.4 ± 0.2 μM). The R2 position includes various groups such as N, F, CF_3_, Br, Cl, and Me, with these substituents exhibiting significant differences in activity. The initial scaffolds included carboxamide linkers, however the scaffolds had limited KDM4B activity. Compound **6** proves to be the exception, with an isonicotinic acid group, it has one of the lowest IC_50_ values, despite the carboxamide linker proving to be less active. Compounds with the fluorine at R2 generally displayed good activity, with some exceptions like compound **18** (IC50 2.3 ± 0.4 μM). Br substitution at R2, seen in compound **10**, enhances potency, suggesting a beneficial effect of bulkier halogens at that position. The R4 position is predominately hydrogen, with non-hydrogen substitutions in compounds **16** and **19** showing no improvement with inhibitory activity, suggesting that H should be maintained at this position.

The R4 position remains consistent across the series with hydrogen aside from compound **6**, which is a naphthyl group, indicating that this position might not play a critical role in affecting activity. Naphthyl, methylphenyl, methoxy, and halogen variations on the second ring significantly influence activity. Compounds **7-9** containing a 4-MePh exhibit moderate to good activity, suggesting this group is favorable. The bulkier groups in compound **15** or more flexible groups in compound **19** generally indicated reduced activity based on the 20 μM screen in Fig. 2.

Potent inhibitors such as compounds **6, 8, 10, 14**, and **21** demonstrate the most potent inhibitory activity, suggesting key structural features that enhance inhibition. Maintaining the carboxylic acid at R1 and exploration of further halogen substituents at R2 may prove to be beneficial. Further optimization of R5 with 4-subsituted phenyl groups, particularly 4-ClPh, should be done to identify its role in enhancing KDM4B inhibitory activity. This SAR analysis provides a starting point to understanding how different substituents and structural variations influence the inhibitory activity of these compounds against KDM4B and allows for guidance in future derivatization. The top compound from the group of active scaffolds is compound **14**, with an IC_50_ of 0.17 ± 0.03.

### Evaluation of enzyme kinetics

We generated a Michaelis-Menten inhibition profile (Supplementary Fig. 1A) and corresponding Lineweaver-Burk plot (Supplementary Fig. 1B) for inhibition with recombinant KDM4B with 0, 0.125, and 0.5 μM **14** with varying concentrations of substrate. Collectively, these data indicate that **14** is a mixed inhibitor of KDM4B. A commercially available secondary assay was used to confirm the results from the bulk histone assay. The assay uses the formation of succinate in the first demethylation step as a bioluminescent readout of demethylase activity. The IC_50_ of control compound **1** and top compound **14** are shown in Supplementary Fig. 2, indicating results within the standard deviation of the bulk histone assay: **Compound 1:** 2.6 ± 0.5 μM, **Compound 14:** 0.13 μM ± 0.03.

### In silico docking experiments

Potential interactions of compound **14** with KDM4B were probed via in silico docking in the catalytic active site using the Molecular Operating Environment (MOE) Dock module to flexibly dock compounds **14** into KDM4B X-ray structure [PDB:4LXL]. These results are depicted in Fig. 2C. The pocket is coordinated around the Fe^2+^ in the binding pocket. The iron moiety interacts with the sulfonamide linker as well as the carboxylic acid group. In this model, compound **14** forms a hydrogen bond with Ile72. Associated interactions within the pocket include Lys207 and the third ring and Ser197 and the carboxylic acid group. Additionally, pi-pi stacking between the aromatic ring of Phe186 with the central aromatic ring of the compound is observed. Ionic interactions are noted between compound **14** and Lys242 positively charged side chain. Several electrostatic interactions with amino acids within the catalytic pocket: Asp136, Glu191, and His189 are also observed. Compound **14** forms additional hydrogen bonds in the pocket and is afforded more interactions due to the presence of a chlorine on the third ring, subtle interactions with Asn379 and His192, and more pronounce hydrophobic interactions.

### Cytokine expression

It has been established that pro-inflammatory cytokines IL-6 and TNF-alpha are upregulated and a critical part of the chronic inflammation in PD [38]. To determine the effects of KDM4B inhibition on macrophage cytokine secretion, cells were treated with 50 μM of compound, stimulated with *A.a*. LPS for 24 h, and cell supernatant was collected for ELISA analysis. After 1-hour incubation with KDM4B inhibitor and the LPS stimulation, ELISA analysis revealed that the exploratory compounds reduced the levels of inflammatory cytokines in murine macrophages. IL-6 response was more robust than TNF-a, exhibiting significant differences from LPS control with several of the top compounds, *6-8, 12, 14*, and control compound *1* (Fig. 3A, B). The compounds demonstrated significantly reduced cytokine expression. IL-6 response with DMSO LPS control was much higher than TNF-α. Compound *14* showed a 137-fold decrease in IL-6 serum concentration and a 7-fold reduction in TNF-α. Compound **14** was further evaluated and determined to exhibit a dose-dependent response in cytokine production (Fig. 3C). TNF-α reduction mirrored this response with compound **14** (Fig. 3D). The IL-6 expression in LPS control was about 20x higher than TNF-α, and the reductions follow the same trend with the highest treatment dose of 100 μM being about 20-fold different. These data demonstrate that the improved potency against KDM4B reflects equal improvements in inflammatory cytokine reduction.

### KDM4B expression and H3K9me3 methylation

To ensure that the cellular response in cytokine reduction is consistent with KDM4B inhibition, we compared KDM4B expression levels and H3K9me3 methylation in murine macrophage RAW264.7 cells. Cells were treated as described above, and after 24H, were fixed and stained using an anti-murine KDM4B PE-conjugated antibody, or an anti-H3K9me3 PE-conjugated antibody and counter-stained with DAPI. The ratio of the KDM4B antibody fluorescent area to DAPI fluorescent area was used as a measure of KDM4B expression and is presented in Fig. 4. It was determined that with LPS stimulation, KDM4B expression increased almost 9-fold, and with treatment, was reduced back 2.7-fold. To determine target validation and mechanism within the macrophage cells, we assessed methylation changes, Raw264.7 cells were treated with **14** or control ML324 at various concentrations for 24 H. Cells were stained with an H3K9me3 PE-conjugated antibody. H3K9me3 levels peaked in a concentration-dependent manner (Fig. 5). These results consistently align with the cytokine reduction, indicating the reduction is KDM4B and H3K9me3 dependent.

### Cell viability

Cell viability was tested after a 24H treatment with experimental compounds. Cell viability was evaluated using the Promega Cell Titer-Glo assay (Promega, Madison, WI). Compounds evaluated included those with potent KDM4B inhibitory activity and diverse structural features, such as compound **14** with the R2 chloro and R6 flouro moieties, compound **6** with a napthyl and isonicotinic acid, and **8** with a tetrazole at R7 (Fig. 6). Compound **14** exhibited an LC50 projected value of 57. 3 ± 23.1 μM. indicating a low degree of toxicity. This cytotoxicity range is favorable and represents an improvement over the millimolar range observed for other KDM4 inhibitors [34]. This data suggest that the proposed compounds do not alter the inflammatory response by compromising cellar integrity but rather by maintaining the repressive H3K9me3 marker, thereby alleviating inflammatory gene expression.

### Osteoclast formation

KDM4B is known to be an epigenetic regulator of osteoclast differentiation. KDM4B associates with CCAR1-med1 complex, which localizes to the promoters of multiple osteoclast-related genes, promoting bone loss [39]. To evaluate the effects of inhibition of KDM4B with our compounds in osteoclastic formation and its potential to reduce bone loss, we differentiated RAW264.7 macrophages into osteoclasts [40]. After 5 days of priming RAW264.7 cells into pre-osteoclasts using receptor activator of nuclear factor kappa B ligand (RANK-L), osteo- clastogenesis was significantly increased in cells treated with RANK-L or Aa-LPS for 3 days compared to PBS treated cells. This phenotype was lost in cells that were pretreated with control compound *1* or **14** (20 μM). TRAP+, multinucleated cells were significantly more numerous in cells treated with RANK-L and Aa LPS compared to PBS treated control cells. Additionally, the formed osteoclasts in the treatment groups appeared smaller and exhibited less intense TRAP staining (Fig. 7A, B). The data depicted is a zoomed in view, with the larger cell area depicted in Supplementary Fig. 3. These data suggest that KDM4B inhibition not only alleviates pro-inflammatory cytokine response but also decreases osteoclastogenesis in murine macrophages.

## Discussion and conclusion

Our study addresses the significant challenges faced in developing small molecule KDM4 inhibitors, specifically achieving potency at low concentrations and adequate cell penetration. To overcome these challenges, we synthesized a library of compounds iteratively designed and tested to identify potent inhibitors with improved characteristics such as cell permeability, solubility, and low cytotoxicity.

The first set of compounds synthesized explored substitution patterns of the aminobenzoic acid moiety and the biphenyl ring system. Initial screening at 20 μM revealed that certain substitution patterns significantly influenced inhibitory activity against KDM4B. Building on these findings, the next set of compounds maintained the active 2-aminobenzoic acid moiety while incorporating additional permutations of biphenyl substitutions. After further evaluation, only a few compounds demonstrated significant KDM4B inhibition, highlighting the need for further optimization. Focusing on enhancing the electronegativity, we modified the linker to sulfonamide benzoic acids and designed compounds with various functional groups such as halogens, methyl, methoxy, and trifluoromethyl moieties. Bioisosteric substitution of the carboxylic acid with tetrazoles and replacement of the carboxamide with sulfonamide were also investigated. This iteration yielded compounds with impressive KDM4B inhibitory activity, particularly compound **14**, which exhibited an IC_50_ of 170 nM. Detailed enzyme inhibition studies indicated mixed inhibition of KDM4B. In silico docking further revealed multiple interactions within the catalytic pocket of KDM4B, including hydrogen bonding, pi-pi stacking, and electrostatic interactions. The presence of a chlorine ring facilitated additional interactions, influencing the electronic distribution of the compound and interactions with amino acids such as Asn87 and Lys242 (Fig. 2C).

The biological evaluation of compound **14** and other top candidates demonstrated significant reductions in pro- inflammatory cytokines IL-6 and TNF-α in the murine macrophage model. This cytokine reduction was consistent with decreased KDM4B expression and increased H3K9me3 methylation, confirming the mechanism of action. Additionally, compound **14** exhibited low cytotoxicity, indicating its potential as a probe for immune modulation. The addition of compound **14** in osteoclast differentiation in pre-osteoclast RAW264.7 cells highlights its potential for use in modulating PD progression.

Compound **14** emerged as a highly potent inhibitor through the iterative design process, supported by preliminary SAR analysis and biological evaluations. These data underscore the potential for KDM4B inhibition in modulating key inflammatory pathways implicated in PD. Given the lack of effective strategies targeting the immune response in PD, these findings provide a promising new approach for the development of therapeutics aimed at modulating the immune response through epigenetic mechanisms.

Future work will focus on further optimization of the identified scaffold, detailed mechanistic studies, exploration of additional analogs to develop a comprehensive SAR profile, and transition into studies using human macrophage cell lines. We anticipate that adjunctive targeting of the epigenetic enzyme KDM4B will be beneficial following SRP treatments and antibacterial therapy. This approach aims to prime the cells by reducing plaque biofilm and bacterial presence, optimizing their response to antiinflammatory stimuli. The synthesis of optimized compounds related to compound **14** and further exploration of KDM4B immune modulation are ongoing in our laboratory, advancing the field of epigenetic based therapies and offering new ways for effective treatment strategies against KDM4B.

### Experimental

#### General procedures

All reagents and dry solvents were purchased from Aldrich Chemical Co. (Milwaukee, WI), Sigma Chemical Co. (St. Louis, MO), VWR (Radnor, PA) or Fisher Scientific (Chicago, IL) and were used without further purification except as noted below. Triethylamine was distilled from potassium hydroxide and stored in a nitrogen atmosphere. Dry methanol, ethyl acetate, tetrahydrofuran, dimethyl formamide and hexane were either purchased (VWR) or prepared using a Glass Contour Solvent Purification System (Pure Process Technology, LLC, Nashua, NH). Preparative scale chromatographic procedures were carried out using a Biotage Selekt chromatography system (Biotage, Charlotte, NC) fitted with silica gel 60 cartridges (230–440 mesh). Thin layer chromatography was conducted on Merck precoated silica gel 60 F-254. Compound CC-90011 and tranylcypromine were purchased from Selleckchem (Houston, TX). All ^1^ H- and ^13^C NMR spectra were recorded on a Bruker Avance 600 MHz spectrometer, and all chemical shifts are reported as d values referenced to TMS or DSS. Splitting patterns are indicated as follows: s, singlet; d, doublet; t, triplet; m, multiplet; br, broad peak. In all cases, 1H NMR, 13 C NMR and MS spectra were consistent with assigned structures, and 13 C peak assignments appear on the spectrum. Mass spectra were recorded by LC/MS on a Waters UPLC/MS system with a model QDa mass spectrometer detector. Prior to biological testing procedures, all compounds were determined to be >95% pure by UPLC chromatography (9:1 H2O: acetonitrile, +0.1% formic acid to 1:9 H2O/acetonitrile +0.1% formic acid over 8 min) using a Waters Acquity H-series ultrahigh-performance liquid chromatograph fitted with a C18 reversed-phase column (Acquity UPLC BEH C18 1.7 M, 2.1 × 100 mm). Compounds 6-21 were synthesized according to the general procedures described below. All data are represented as geometric mean ± standard error of the mean (SEM) and statistical significance was determined using One-Way ANOVA with multiple comparisons (p < 0.05).

### Synthetic Chemistry

#### Method A: Compounds 9-21

The method for synthesizing the Arylsulfonyl chlorides is as previously described [36]. (1.0 eq) and methyl 2-aminobenzoate (1.0 eq) were dissolved in anhydrous dichloromethane and stirred for 10 minutes, at which time pyridine (1.0 eq) was added dropwise. The reaction was allowed to proceed with stirring at room temperature for 12 h. After completion, as determined by TLC, the reaction was acidified to pH 4 with 1 M HCl, and the product was extracted with dichloromethane. The combined organic phases were washed with brine, dried over Na2SO4, and filtered. Solvent was then removed under reduced pressure, and the crude reaction mixture was purified by trituration using dichloromethane and hexanes. To a stirred solution of product methyl 2-(arylsulfonamido)benzoate in tetrahydrofuran, 1 M aqueous LiOH (2 eq) was added. The reaction was allowed to proceed with stirring at room temperature overnight. Solvent was then evaporated under reduced pressure, and the residue was subsequently diluted with water and acidified to pH 2 using 1 M HCl. The mixture was then extracted using EtOAc, and the combined organic phases were washed with brine, dried over Na2SO4, and filtered. the solvent was then removed under reduced pressure to provide the corresponding 2-(arylsulfonamido)benzoic acid as an off-white solid. The product was purified by normal phase flash chromatography using a dichloromethane/methanol mobile phase. Products needing additional purification were recrystallized from ethyl acetate and hexanes.

#### Method B: Compound 8

The synthesis is as previously described [36]. Arylsulfonyl chloride (1.0 *eq*) and methyl 2-aminobenzonitrile (1.0 *eq*) were dissolved in anhydrous dichloromethane and stirred for 10 minutes, at which time pyridine (1.0 eq) was added dropwise. The reaction was allowed to proceed with stirring at room temperature for 12 h. After completion, as determined by TLC, the reaction was acidified to pH 5 with HCl, and the product was extracted with dichloromethane. The combined organic phases were washed with brine, dried over Na2SO4, and filtered. Solvent was then removed under reduced pressure, and the crude reaction mixture was purified by trituration using dichloromethane and hexanes. To a stirred solution of product 2-(arylsulfonamido)benzonitrile (1.0 *eq*) in DMSO, sodium azide (1.0 *eq*) and copper(II) sulfate (0.02 *eq*) were added. The reaction was heated to 150 °C and allowed to proceed with stirring for 1 h. After cooling, the reaction mixture was diluted with water and acidified to pH 2 using 1 M HCl. The mixture was then extracted using EtOAc, and the combined organic phases were washed with brine, dried over Na2SO4, and filtered. The solvent was then removed under reduced pressure to provide the corresponding *N*-(*2*-(*1H*-tetrazol-5-yl)phenyl)benzenesulfonamide as an off-white solid. The product was purified by normal phase flash chromatography using a dichloromethane/methanol mobile phase. Products needing additional purification were recrystallized from ethyl acetate and hexanes.

#### Method C: Compound 6

2-napthoyl chloride (1.0eq) and methyl-3aminoisonicotinate (1.0eq) was dissolved in anhydrous dichloromethane and stirred for 10 minutes, at which time pyridine (1.0eq) was added dropwise. The reaction was allowed to proceed with stirring at room temperature for 12 h. After completion as determined by TLC, the reaction was acidified to pH 5 with HCl, and the product was extracted with DCM. The combined organic phases were washed with brine, dried over Na2SO4, and filtered. Solvent was then removed under pressure, and the crude reaction mixture was purified by titration using dichloromethane and hexanes. TO a stirred solution of product methyl 3-(2-napthamido)isonicotinate (1.0eq) in methanol water 3:1 ratio, 1 M LiOH (2eq) was added. The reaction was allowed to proceed with stirring at room temperature overnight. Solvent was then evaporated under reduced pressure, and the residue was subsequently diluted with water and acidified to pH 2 using 1 M HCl. The mixture was then extracted using EtOAc, and the combined organic phases were washed with brine, dried over Na2SO4, and filtered. Solvent was then removed under reduced pressure to provide the corresponding 3-(2-napthamido)isonicotinic acid as an off-white solid. The product was purified by normal phase flash chromatography using a dichloromethane/methanol mobile phase. Products needing additional purification were recrystallized from ethyl acetate and hexanes.

#### Compound analysis

##### Compound 6: 3-(2-naphthamido)isonicotinic acid (6)

Purity 98.61% ^1^H NMR (C_2_D_6_OS, 600 MHz): δ 7.67 (d, J = 4.86 Hz, 1 H) 7.87 (d, J = 4.62 Hz, 1 H) 8.04 (d, J = 19.86 Hz, 1 H) 8.12 (t, J = 8.67 Hz, 1 H) 8.50 (d, J = 4.38 Hz, 1 H) ^13^C NMR (C_2_D_6_OS, 150 MHz): δ 101.54, 101.70, 102.70, 102.85, 105.29, 105.46, 107.20, 110.80, 110.94, 113.61, 122.08, 128.34, 128.39, 129.19, 129.91, 132.04, 134.55, 134.62, 134.86, 134.93, 135.02, 135.71, 142.53, 154.11, 154.19, 164.55, 166.22, 169.24, 169.53 ESI-MS for C_17_H_12_N_2_O_2_: [M-H]- calculated 276.30, found 275.45

##### Compound 7: 4,5-difluoro-2-((4’-methyl-[1,1’-biphenyl])-4-sulfonamido)benzoic acid (7)

Purity 94.9% ^1^H NMR (C_2_D_6_OS, 600 MHz): δ 2.34 (s, 3H), 7.30 (d, 2H, *J* = 7.9 Hz), 7.51 (d, 1H, *J* = 6.4 Hz), 7.61 (d, 2H, *J* = 8.1 Hz), 7.86 (m, 5H, *J* = 9.5 Hz), 11.29 (br, 1H).^13^C NMR (C_2_D_6_OS, 150 MHz): δ 20.6, 108/0, 108.1, 120.0, 126.9, 127.3, 127.5, 129.7, 135.0, 136.6, 138.3, 145.0, 167.9. ESI-MS for C_20_H_15_F_2_NO_4_S: [M-H]- calculated 402.1, found 402.2.

##### Compound 8: N-(4-fluoro-2-(1H-tetrazol-5-yl)phenyl)-4’-methyl-[1,1’-biphenyl]-4-sulfonamide (8)

Purity 96.78% ^1^H NMR (C_2_D_6_OS, 600 MHz): δ 2.28 (d, 6H) (d, 1 H) 7.23 (d, 2 H) 7.31 (d, 1 H) 7.41 (d, 1 H) 7.51 (d, 2 H) 7.66 (d, 5 H) 10.41 (d, 1 H) ^13^C NMR (C_2_D_6_OS, 150 MHz): δ 144.99, 138.72, 135.63, 130.13, 127.86, 127.37, 127.31, 122.59, 115.79, 21.13, 20.64 ESI-MS for C_20_H_16_FN_5_O_2_S: [M-H]- calculated 409.1, found 408.2.

##### Compound 9: 2-((4’-methyl-[1,1’-biphenyl])-4-sulfonamido)-5-(trifluoromethyl)benzoic acid (9)

Purity 99.75% ^1^H NMR (C_2_D_6_OS, 600 MHz): δ 11.86 (brs, 1H); 8.13 (d, 1H, J = 1.92 Hz); 7.94 (d, 2H, J = 8.58 Hz); 7.88 (dd, 1H, J = 2.01, 8.8 Hz); 7.84 (d, 2H, J = 8.64 Hz); 7.6915 (d, 1H, J = 8.76 Hz); 7.5895 (d, 2H, J = 8.16 Hz); 7.2726 (d, 1 =2H, J = 7.98 Hz); 2.32 (s, 3H). ^13^C NMR (C_2_D_6_OS, 150 MHz): δ 169.05, 145.54, 143.88, 138.88, 137.50, 135.56, 131.45, 130.22, 128.85, 128.06, 127.91, 127.42, 118.62, 117.38, 21.17. ESI-MS for C_21_H_16_F_3_NO_4_S: [M-H]- calculated 435.4, found 435.3.

##### Compound 10: 5-bromo-2-((4’-methyl-[1,1’-biphenyl])-4-sulfonamido)benzoic acid (C_20_H_16_BrNO_4_S) (10)

Purity 94.8% ^1^H NMR (C_2_D_6_OS, 600 MHz): δ 11.13 (brs, 1H); 7.95 (d, 1H, J = 2.5 Hz;); 7.84 (q, 4H, J = 8.8 Hz); 7.73 (dd, 1H, J=2.5 and 8.8 Hz); 7.58 (d, 2H, J = 8.2 Hz); 7.48 (d, 1H, J = 8.9 Hz); 7.27 (d, 2H, J = 7.9 Hz); 2.32 (s, 3H).

^13^C NMR (C_2_D_6_OS, 150 MHz): δ 168.8, 145.4, 139.4, 138.9, 137.45, 137.3, 135.6, 134.1, 130.2, 128.0, 127.8, 127.4, 121.1, 119.6, 115.5, 21.2. ESI-MS for C_20_H_16_BrNO_4_S: [M-H] – 445.00 calculated found 445.2.

##### Compound 11: 5-chloro-2-((4’-methyl-[1,1’-biphenyl])-4-sulfonamido)benzoic acid (11)

Purity 96.1% ^1^H NMR (C_2_D_6_OS, 600 MHz): δ 2.34 (s, 3H), 7.29 (d, 2H, *J* = 8.0 Hz), 7.56 (d, 1H, *J* = 8.9 Hz), 7.60 (d, 2H, *J* = 4.2 Hz), 7.62 (d, 1H, *J* = 2.6 Hz), 7.83 (d, 2H, *J* = 8.9), 7.85 (m, 1H), 7.86 (d, 2H, *J* = 8.6 Hz), 11.50 (br, 1H). ^13^C NMR (C_2_D_6_OS, 150 MHz): δ 20.6, 119.2, 120.3, 126.9, 127.0, 127.2, 127.4, 129.7, 130.6, 133.9, 135.1, 137.0, 138.3, 138.7, 144.8, 168.3. ESI-MS for C_20_H_16_ClNO_4_S: [M-H]- calculated 400.0, found 400.2, 402.2.

##### Compound 12: 5-fluoro-2-((3’-methyl-[1,1’-biphenyl])-4-sulfonamido)benzoic acid (12)

Purity 98.30% ^1^H NMR (C_2_D_6_OS, 600 MHz): δ 10.84 (bs, 1H); 7.80 (ps, 4H); 7.60 (dd, 1H, J = 4.1 and 5.9 Hz); 7.56 (dd, 1H, J = 4.8 and 9.2 Hz); 7.47 (ps, 1H); 7.44 (d, 2H, J = 8.7 Hz); 7.33 (t, 1H, J = 7.6 Hz); 7.20 (d, 2H, J = 7.5Hz) ^13^C NMR (C_2_D_6_OS, 150 MHz): δ 168.86, 158.86, 157.25, 145.51, 138.84, 138.47, 129.81, 129.46, 128.17, 128.00, 127.93, 124.65, 122.23, 122.18, 122.03, 120.18, 120.13, 117.95, 117.79, 21.45. ESIMS for C_20_H_16_FNO_4_S: [M-H]- calculated 385.4, found 384.2

##### Compound 13: 5-fluoro-2-((4’-methoxy-[1,1’-biphenyl])-4-sulfonamido)benzoic acid (13)

Purity 98.36% ^1^H NMR (C_2_D_6_OS, 600 MHz): δ 10.88 (brs, 1H); 7.78 (dd, 2H, J = 2.8 and 18.1 Hz); 7.65 (d, 2H, J = 8.8 Hz); 7.60 (dd, 2H, J = 2.18 and 6.0 Hz); 7.55 (dd, 2H, J = 4.80 and 9.1 Hz); 7.45 (dt, 2H, J = 3.1 and 8.0 Hz)7.02 (dd, 2H, J = 8.8 Hz); 248 (s, 3H). ^13^C NMR (C_2_D_6_OS, 150 MHz): δ 168.86, 160.38, 158.80, 157.19, 145.00, 136.83, 136.39, 130.66, 128.81, 127.96, 127.29, 122.10, 122.05, 121.88, 120.11, 117.94, 117.78, 115.05, 55.74. ESI-MS for C_20_H_16_FNO_5_S: [M-H]- calculated 401.4, found 400.3

##### Compound 14: 2-((4’-chloro-[1,1’-biphenyl])-4-sulfonamido)-5-fluorobenzoic acid (14)

Purity 98.88% ^1^H NMR (C_2_D_6_OS, 600 MHz): δ 10.89 (brs, 1H); 7.83 (d, 4H, J = 7.8 Hz); 7.71 (d, 2H, J = 8.5 Hz); 7.60 (dd, 1H, J = 3.0 and 9.1 Hz); 7.55 (dd, 1H, J = 4.8Hz) 7.52 (d, 2H, J = 8.4 HZ); 7.45 (dt, 1H, J = 2.9 and 17.0 Hz) ^13^C NMR (C_2_D_6_OS, 150 MHz): δ 168.85, 158.92, 156.73, 144.00, 138.06, 137.32, 136.23, 134.19, 129.57, 129.39, 128.07, 128.03, 122.28, 122.23, 122.00, 121.85, 117.96, 117.80. ESI-MS for C_19_H_13_ClFNO_4_S: [M-H]- calculated 405.8, found 404.7.

##### Compound 15: 5-fluoro-2-(naphthalene-2-sulfonamido)benzoic acid (15)

Purity 96.82% ^1^H NMR (C2D6OS, 600 MHz): δ 8.40 (ps, H); 8.03 (dd, 2H, J = 8.5 and 18.0 Hz); 7.94 (d, 2H, J = 8.1 Hz); 7.65 (t, 2H, J = 8.0 Hz); 7.61 (t, 2H, J = 7.5 Hz); 7.55 (dd, 2H, J = 4.7 and 9.1 Hz); 7.49 (dd, 2H, J = 2.91 and 8.9 Hz); 7.34 (dt, 2H, J = 2.9 and 8.4 Hz). 13C NMR (C2D6OS, 150 MHz): δ 168.81, 163.86, 162.23, 158.83, 157.22, 144.25, 137.78, 136.36, 135.01, 129.79, 129.74, 128.00, 127.97, 122.17, 122.12, 121.94, 121.79, 120.40, 120.35, 117.93, 117.77, 116.53, 116.39. ESI-MS for C17H12FNO4S: [M-H]- calculated 345.4 found 344.3.

##### Compound 16: 2-((4-cyclohexylphenyl)sulfonamido)benzoic acid (15)

Purity 100.00% 376.3 ^1^H NMR (C_2_D_6_OS, 600 MHz): δ 10.23 (brs, ^1^H); 7.65 (d, 2H, J = 8.4 Hz); 7.58 (dd, ^1^H, J = 3.5 and 1.5 Hz); 7.51 (dd, ^1^H, J = 9.1 and 4.8 Hz); 7.43 (dt, ^1^H, J = 8.0 and 3.1 Hz); 7.38 (d, 2H, J = 8.4Hz) 2.53 (m, ^1^H); 1.72 (m, 4H); 1.65 (psd, ^1^H); 1.32 (m, 4H); 1.18 (m, ^1^H) ^13^C NMR (C_2_D_6_OS, 150 MHz): δ 136.61, 128.21, 127.40, 121.79, 43.95, 33.82, 26.55, 25.85 ESI-MS for C_19_H_21_NO_4_S: [M-H]- calculated 377.43 found 376.4.

##### Compound 17: 2-((4’-(trifluoromethyl)-[1,1’-biphenyl])-4-sulfonamido)benzoic acid (17)

Purity 94.55% ^1^H NMR (C_2_D_6_OS, 600 MHz): δ 6.74 (dd, J = 4.80, 9.12 Hz, 1 H) 7.12 (d, J = 5.88 Hz, 1 H) 7.12 (d, J = 3.18 Hz, 1 H) 7.34 (d, J = 3.18 Hz, 1 H) 7.36 (d, J = 3.12 Hz, 1 H) 7.44 (d, J = 3.12 Hz, 1 H) 7.46 (d, J = 2.10 Hz, 1 H) 7.47 (d, J = 3.12 Hz, 1 H) 7.55 (d, J = 4.74 Hz, 1 H) 7.56 (d, J = 4.80 Hz, 1 H) 7.59 (d, J = 3.12 Hz, 1 H) 7.61 (d, J = 3.12 Hz, 1 H) 7.69 (d, J = 7.86 Hz, 1 H) 7.78 (d, J = 7.62 Hz, 1 H)7.84 (d, J = 16.38 Hz, 1 H) ^13^C NMR (C_2_D_6_OS, 150 MHz): δ 168.79, 157.30, 143.71, 142.57, 138.77, 136.18, 128.59, 128.50, 128.06, 126.40, 122.34, 117.96, 117.80 ESI-MS for C_20_H_14_F_3_NO_4_S: [M-H]- calculated 439.8, found 438.4.

##### Compound 18: 5-fluoro-2-((4’-fluoro-[1,1’-biphenyl])-4-sulfonamido)benzoic acid (18)

Purity 96.83% ^1^H NMR (C_2_D_6_OS, 600 MHz): δ 11.01 (brs, ^1^H); 7.81 (psd, 4H, J = 1.54 Hz); 7.74 (dd, 2H, J = 4.7 and 5.4 Hz); 7.60 (dd, 1H, J = 3.1 and 6.0 Hz); 7.55 (dd, 1H, J = 4.3 and 4.6 Hz); 7.45 (dd, 1H, J = 3.1 and 8.0 Hz); 7.30 (t, 2H, J = 8.8 Hz). ^13^C NMR (C_2_D_6_OS, 150 MHz): δ 168.81, 163.86, 162.23, 158.83, 157.22, 144.25, 137.78, 136.36, 135.01, 129.79, 129.74, 128.00, 127.97, 122.17, 122.12, 121.94, 121.79, 120.40, 120.35, 117.93, 117.77, 116.53,116.39. ESI-MS for C_19_H_13_F_2_NO_4_S: [M-H]- calculated 389.4, found 388.3.

##### Compound 19: 5-fluoro-2-((5,6,7,8-tetrahydrona phthalene)-2-sulfonamido)benzoic acid (19)

Purity 92.37% ^1^H NMR (C_2_D_6_OS, 600 MHz): δ 10.78 (brs, 1H); 7.60 (dd, 1H, J=3.0 and 9.0 Hz); 7.50 (dd, 1H, J=4.8 and 9.1 Hz); 7.46 (bs, 1H); 7.44 (dd, 1H, J = 2.6 and 8.6 Hz); 7.42 (d, 1H, J = 7.68 Hz); 7.19 (d, 1H, J = 8.04 Hz); 2.70 (m, 4H);1.67 (m, 4H). ^13^C NMR (C_2_D_6_OS, 150 MHz): δ168.95, 158.64, 157.03, 143.63, 138.71, 136.62, 135.98, 130.42, 127.66, 124.10, 122.04, 121.89, 121.78, 121.73, 119.68, 119.64, 117.87, 117.71, 29.19, 29.02, 22.52, 22.43. ESI-MS for C_17_H_16_FNO_4_S: [M-H]- calculated 349.4, found 348.3.

##### Compound 20: 2-((2,3-dihydrobenzo[b][1,4]dioxine)-6-sulfonamido)-5-fluorobenzoic acid (20)

Purity 99.29% ^1^H NMR (C_2_D_6_OS, 600 MHz): δ 4.26 (d, J= 12.48 Hz, 1 H) 4.26 (dd, J = 3.21, 19.59 Hz, 1 H) 6.97 (d, J = 7.80 Hz, 1 H) 6.97 (d, J= 11.40 Hz, 1 H) 6.97 (d, J = 7.86 Hz, 1 H) 7.45 (d, J = 4.14 Hz, 1 H) 7.45 (d, J = 5.94 Hz, 1 H) 7.45 (d, J= 11.04 Hz, 1 H) 7.50 (d, J = 4.80 Hz, 1 H) 7.52 (d, J = 4.86 Hz, 1 H) 7.60 (d, J= 12.18 Hz, 1 H) 7.60 (d, J = 6.00 Hz, 1 H) ^13^C NMR (C_2_D_6_OS, 150 MHz): δ 168.88, 148.20, 143.87, 136.51, 131.14, 122.00, 118.26, 117.87, 117.71, 116.25, 64.87, 64.48 ESI-MS for C_14_H_12_FNO_6_S: [M-H]- calculated 353.04 found 352.1

##### Compound 21: 2-((4’-chloro-[1,1’-biphenyl])-4-sulfonamido)-5-methylbenzoic acid (21)

Purity 99.47% ^1^H NMR (C_2_D_6_OS, 600 MHz): δ 2.21 (d, J= 12.48 Hz, 6 H) 7.36 (dd, 1 H) 7.44 (d 1 H) 7.52 (d,J= 11.40 Hz, 2H) 7.67 (d, J = 7.86 Hz, 1 H) 7.71 (d, J = 4.14 Hz, 2 H) 7.83 (d, 4 H) 10.98 (d, 1 H) ^13^C NMR 39.7, 119.34, 128.01, 128.06, 132.01. ESI-MS for C_20_H_16_ClNO_4_S: [M-H]- calculated 401.05 found 400.1.

### In vitro periodontal disease inflammation model

BMDMs were serum starved for 5-8 h by replacing complete media with media containing only 2% FBS. Cells were then pre-treated with 50μM ML324 (SelleckChem, Cat# S7296), 50 μM experimental inhibitors or DMSO vehicle control for 1 hour in complete media. Pre-treated macrophages were challenged with *A.a* LPS for 24 hours where supernatant protein was analyzed using DuoSet IL-6 ELISA kit (R&D Systems, Cat# DY410). Purity of experimental inhibitors was confirmed to be >95% prior to in vitro evaluation via UPLC analysis.

### Raw264.7 cell culture

Raw264.7 cells were cultured in Dulbecco’s modified essential medium (DMEM) (Corning, Cat#10–013-CV) supplemented with 10% fetal bovine serum (FBS) and 1% penicillin/streptomycin(P/S). Cells were grown to 80% confluence, then pre-treated with ML324 (Selleck Chem, Cat# S7296, 50 μM), compound (50 μM) or DMSO vehicle control. DMSO remained constant in each group at a level of 0.1% to eliminated interference. Cells were challenged with A.a. LPS(10 ng/mL) or PBS vehicle control for 24 hours and media was collected for cytokine analysis.

### Osteoclast formation and TRAP staining

RAW264.7 cells were seeded in a 96-well culture plate (1.5×10^5 cells/mL). After culturing overnight, the medium was replaced with 200uL of alpha-minimum essential medium containing 10% FBS and 50 ng/mL RANKL for 2 days. On day 3, wells were rinsed to remove RANK-L and replacement media was supplemented with or without Aa-LPS (100 ng/ mL) or RANK-L, and ML324 (Seleck Chem, Cat# S7296), test compound, or DMSO vehicle control for 72 hours. Cells were rinsed twice, fixed with 10% glutaraldehyde (Fisher, Cat#O2957-1) and stained for tartrate resistance acid phosphatase (TRAP) using a commercially available kit as described (Fisher Scientific, Cat# NC2256842). Subsequently, 3 representative images per well were captured using an Biotek Cytation 5 microscope. Osteoclasts were quantified as multinucleated, TRAP+ cells and statistically significant differences were computed using a paired one-way ANOVA with multiple comparisons with an alpha of 0.5.

### KDM4B CTH ELISA

An immunoassay was developed to test KDM4B enzyme activity with the synthesized inhibitors. The assay used as described in substrate core histones purified from calf thymus (CTH type II-A; Sigma-Aldrich) and quantified the status of H3K9 tri-methylation. First, CTH was diluted into coating buffer (100 mM sodium carbonate, pH 8.0) to a final concentration of 0.02 mg/mL. Wells of a 96-well high binding plate were then coated with 1 mg CTH and incubated overnight at 4 °C while shaking. Wells designated as blanks were coated with buffer alone (100 nM sodium carbonate pH 8). The following day, plates were blocked for 2 h at RT with blocking buffer (5% w/v) bovine serum albumin (BSA, VWR, Tissue Culture Grade) in phosphate- buffered saline (PBS) and washed 4 times for 5 min at 25 °C with ELISA wash buffer PBST (PBS plus 0.1% v/v Tween 20). Wells were then conditioned by washing twice with KDM reaction buffer (20 mM HEPES, pH 7.5, plus 0.01% v/v Tween 20). Meanwhile, KDM reaction components were prepared as 2-fold concentrated solutions containing KDM4B (HEPES, Fe(NH_4_)_2_(SO_4_)_2_, sodium ascorbate, Tween 20). Concentrated enzyme mixtures were prediluted into water (100 μL 2-fold enzyme solution plus 46 μL H_2_O for each concentration of inhibitor tested). Inhibitor solutions dissolved in 100% DMSO were then added to enzyme mixtures (4 μL to each dilution of enzyme described above) and incubated on ice for 10 min. Pure DMSO was added to the enzyme mixtures in parallel as a vehicle control. Enzymes were finally activated after the 10 min incubation by a 1:4 dilution of a 4-fold solution containing concentrated α-KG. Final enzyme mixtures (200 mL) comprised: 100 nM KDM4 iso-form in 20mM HEPES, pH 7.5; 10 μM Fe(NH_4_)_2_(SO_4_)_2_; 1 mM sodium ascorbate; 1 mM a-KG; and 0.01% (v/v) Tween 20. When applicable, DMSO was present at 2% (v/v). Demethylation reactions were initiated by pipetting, in triplicate, 50 mL of activated KDM4 solutions into appropriate wells of the CTH-coated plate. Demethylation reaction was carried out for 2 h at 37 °C. Plates were then washed 4 times with PBST containing 1% (w/v) BSA (PBST-BSA). Levels of H3K9me3 were detected by addition of primary antibody (rabbit, anti-human pAb to H3K9me3, Invitrogen, #49-1008) diluted 1:1000 into blocking buffer and incubation of the plate for 60 min at 25 °C. Plates were then washed 4 times with PBST-BSA before adding horseradish peroxidase (HRP)–coupled secondary antibody (mouse, anti-rabbit IgG, CST, #7074), diluted 1:2000. Plates were incubated for 60 min at 25 °C, washed 4 times for 5 min with PBST-BSA, and developed for 20 min (or until color saturation) at 25 °C by adding ELISA TMB substrate. Finally, reactions were quenched with 0.2 M H_2_SO_4_ before reading the absorbance at 450 nm with a plate reader. Raw values were corrected for background absorbance from blank wells, and the percentage of H3K9me3 remaining was expressed relative to wells containing 1 mg CTH with the DMSO control. Data were analyzed with GraphPad Prism 9.2.0 using a nonlinear regression model.

### Evaluation of H3K9me3 in RAW264.7 macrophage cells

Cells were fixed with 4% paraformaldehyde for 10 minutes at 25 °C. Following fixation, cells were permeabilized with 0.1% Triton-100 for 10 min at 25 °C. Cells were washed three times with PBS between fixation and permeabilization steps and centrifuged at 800 x g for 5 minutes. Cells were blocked using 1% BSA in PBS (BSA, VWR, Tissue Culture Grade) for 30 min at 25°C. Cells were incubated with antibody (Abcam – H3K4me3, Waltham, MA) at a 1:2,000 dilution and incubated overnight at 4°C. Cells were washed with PBS, centrifuged at 800 x g for 5 min, and counterstained with DAPI at a 1:10,000 dilution for 15 min. Cells were washed 3 times prior to plating. Cells were resuspended in PBS and plated a density of 2 × 105 cells/well. Cells were imaged at 20x (9 fields/well) using a BioTek Cytation 5 imager. The ratio of the integrated sum of fluorescent area of the green channel (anti-H3K4Me2 antibody) to the integrated sum of the blue channel (DAPI) was calculated.

### CTG Assay to determine cytotoxicity

Primary bone marrow cells were plated in clear-bottom, white, 96-well plates at a confluency of 50,000 cells/well in Alpha-MEM+ 10% FBS+ 100 IU/mL Penicillin + 100 μg/mL streptomycin. A 2x working solution of compound (for single treatments of 6 or 48) were prepared in cell medium (DMSO working concentration 0.1%) and then serially diluted. Final DMSO concentration in cell treatments was 0.05%. Cells were incubated for 24 hours at 37 °C, 5% CO2. Cell viability was determined using a Promega Cell Titer-Glo commercially available assay kit (Cell Titer-Glo(R) 2.0 Assay, Catalog No. G9242, Promega, Madison, WI). Equivalent volume of Cell Titer-Glo solution was added to culture medium and incubated for 5 minutes. Luminescence was measured using a Molecular Devices SpectraMax iD3 plate reader. Cell count was done using a brightfield image prior to CTG addition using a BioTek Cytation 5 imager, and fluorescence measured was normalized to cell count in each well due to biologic variability in the primary cell culture. Experiments were done with biological and technical triplicates.

### Molecular docking

Modeling, simulations and structural visualizations were performed using MOE 2019 (Chemical Computing Group, Inc.) Docking simulations were based on PDB file 4LXL. The protein was protonated at T = 310 K, pH 7.0, salt at 200 mM using GB/VI electrostatics. Ligands were also protonated at pH7.0. Docking simulations used a flexible receptor and flexed the ligand, targeting the active site defined by the ligand. For each docking simulation initial placement calculated 50 poses using triangle matching with London dG scoring. The top five poses were then refined using forcefield (Amber10: ETH) and Affinity dG scoring (Escore2). The top pose was used for further analysis.

## Supplementary Material

Supplemental

The online version contains supplementary material available at https://doi.org/10.1007/s00044-024-03362-5.

## Figures and Tables

**Fig. 1:**
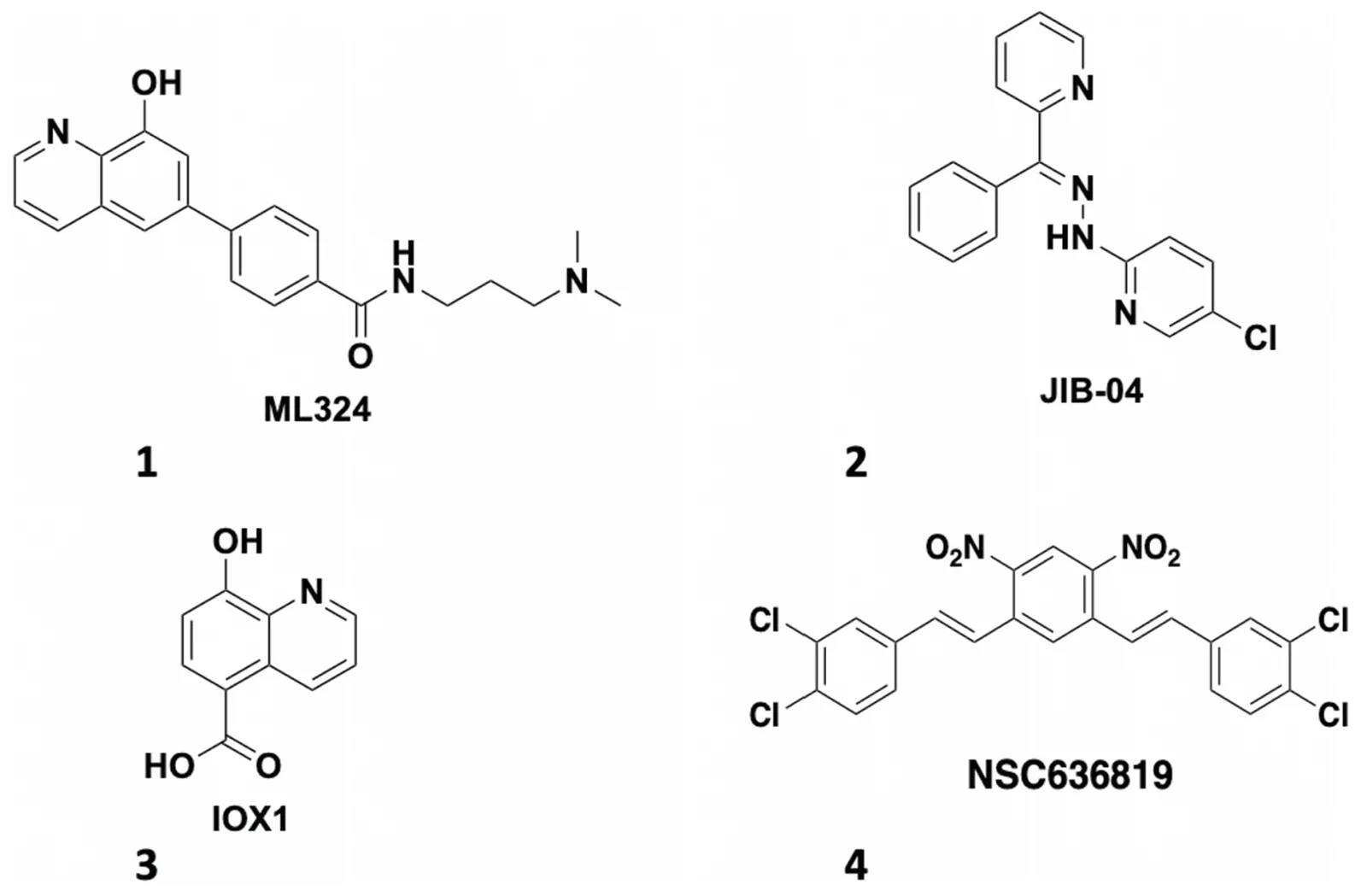
Compounds known to interact with KDM4B.

**Fig. 2:**
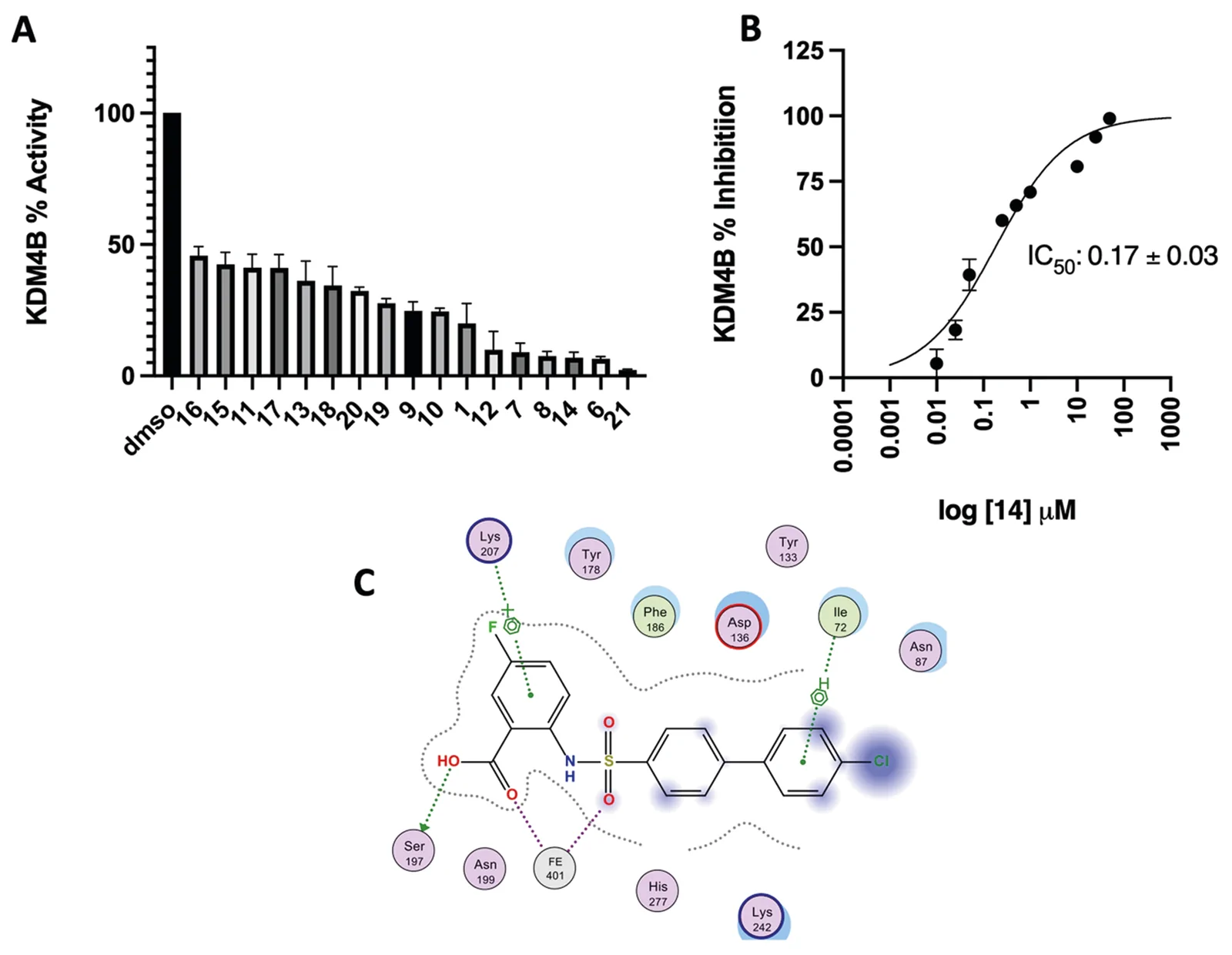
Inhibition of KDM4B and in silico analysis. **A:** 20 μM Screen with compounds 6-21. **B** IC_50_ determination via CTH histone ELISA for compound **14** against recombinant KDM4B.Each data point is the result of three independent determinations ± SEM. **C** Compound 14 flexibly docked with KDM4B starting with X-ray coordinates from PDB:4LXL. KDM4B is grey, Fe(II) is docked in the active site. Molecular interactions are indicated by dashed lines and colored circles represent interactions

**Fig. 3:**
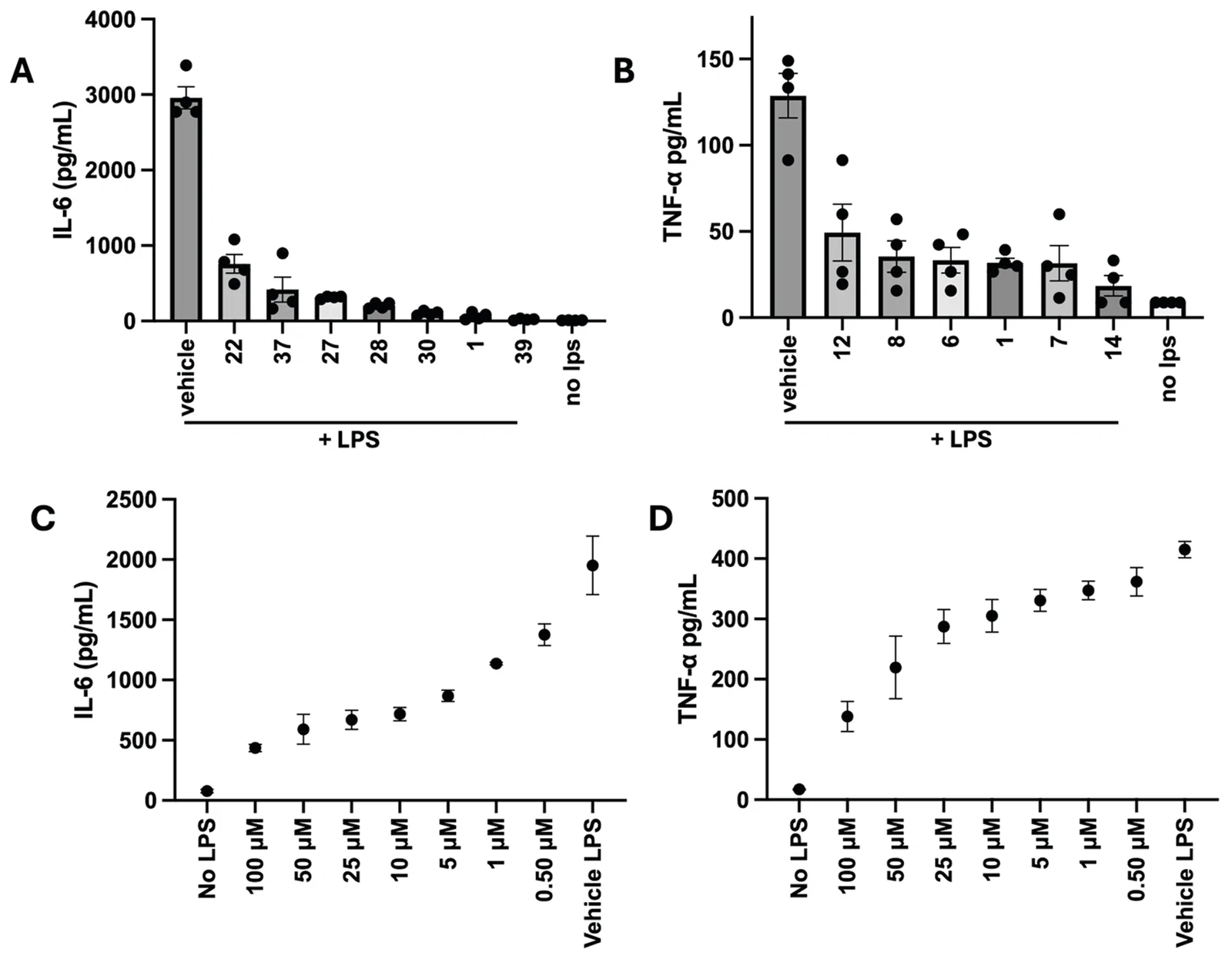
Inhibition of KDM4B causes dose dependent immunosuppression. Cells were treated with compound for 1H prior to LPS stimulation and supernatant was collected after 24H. **A** IL-6 concentration in cell supernatant with and without treatment (20μM) and LPS stimulation. **B** TNF-alpha concentration in cell supernatant with and without treatment and Aa LPS stimulation. **C** Dose Dependent IL-6 response with compound **14. D** Dose dependent TNF-alpha response with compound **14**. Each data point is the result of three independent determinations ± SEM

**Fig. 4:**
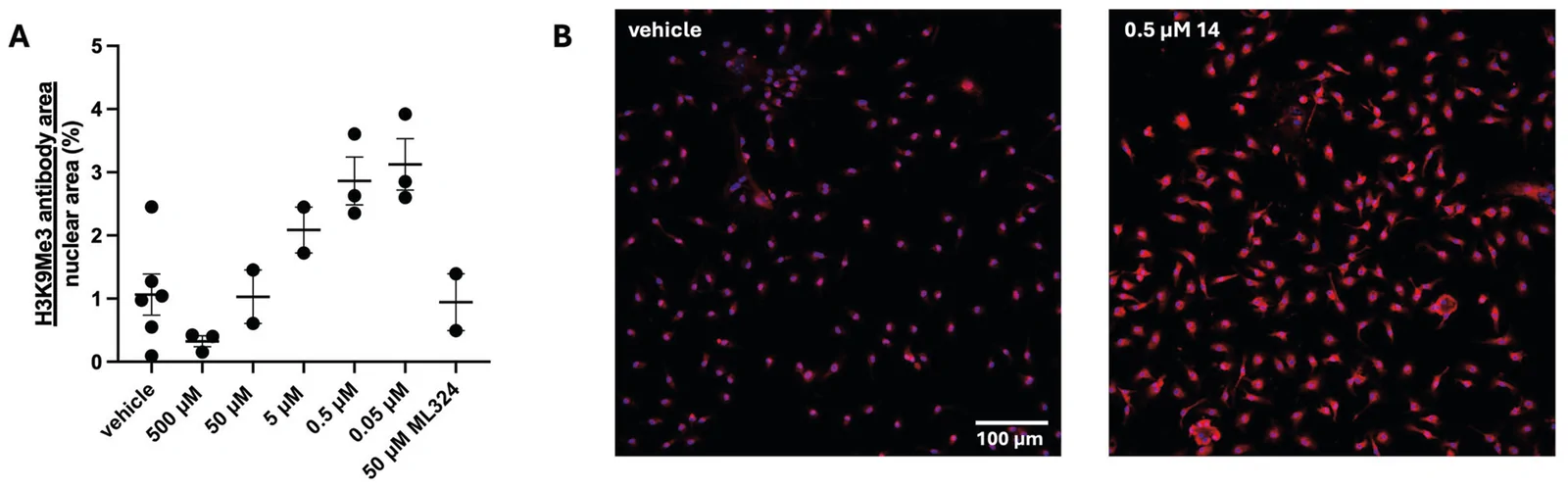
H3K9Me3 antibody fluorescent area:nuclear area for RAW264.7 cells treated with 14. Cells were treated with compound for 1 hour prior to LPS stimulation and analyzed after 24 h. Cells were fixed, permeabilized, and stained with an H3K9me3 antibody containing PE fluorophore. Cells were counterstained with DAPI and imaged at 20X magnification on a Biotek Cytation 5. **A** Quantification of H3K9me3 antibody area:nuclear area yields dose-dependent methylation changes. **B** Untreated cells, Cells treated for 24H with 20 μM **14**. The ratio of H3K9me3 antibody fluorescent area: DAPI fluorescent area was used to measure relative levels of H3K9me3. Each concentration was done as three independent determinations ± SEM

**Fig. 5:**
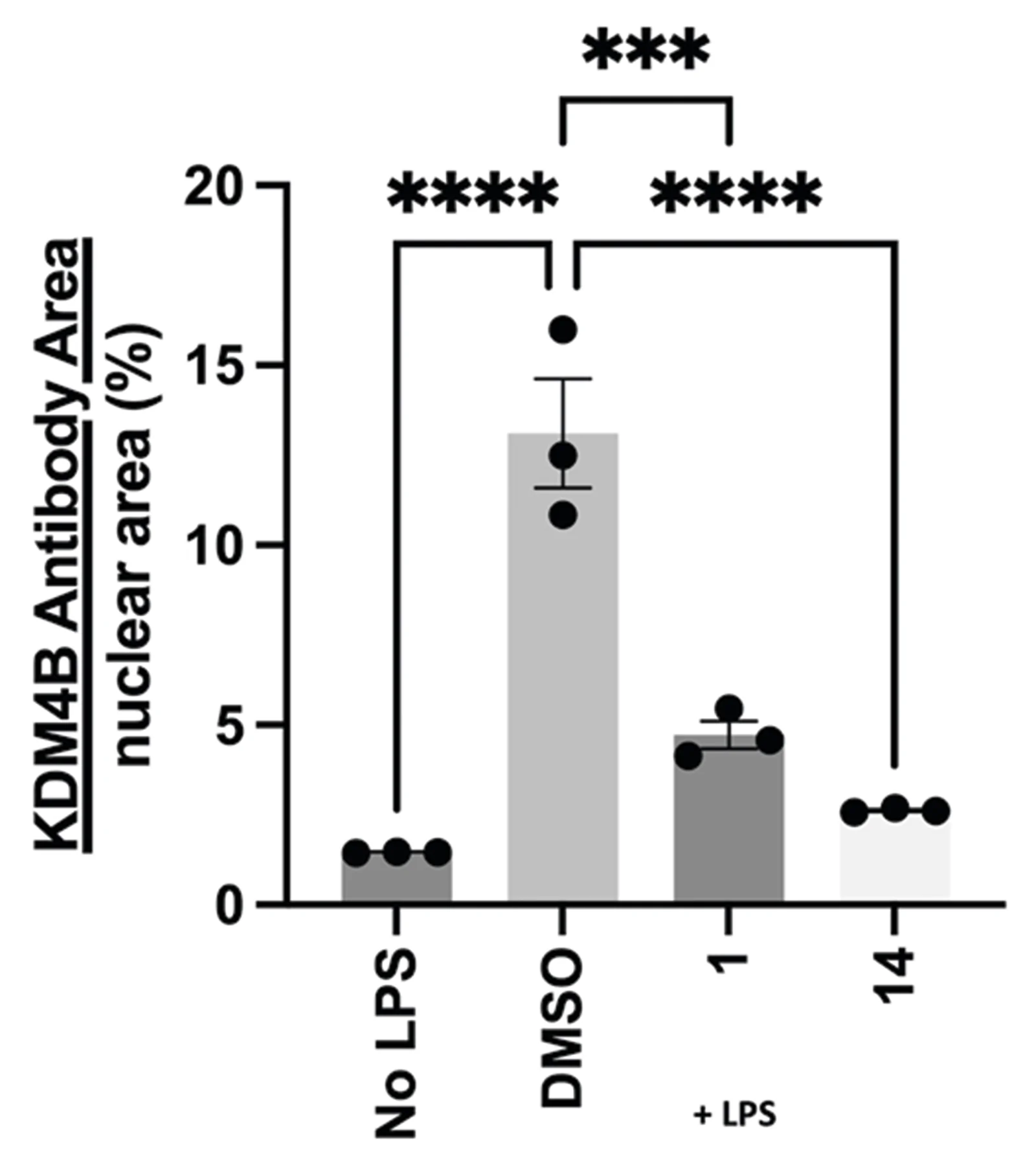
Comparison of KDM4B antibody fluorescent area/nuclear area (%). Cells were fixed and permeabilized and stained KDM4B antibody containing a PE fluorophore. Cells were counterstained with DAPI and imaged at 20x magnification on a Biotek Cytation 5. The ratio of KDM4B fluorescent area: DAPI fluorescent area was used to measure relative levels of KDM4B. Each data point is the result of three independent determinations SEM and *P < 0.01

**Fig. 6:**
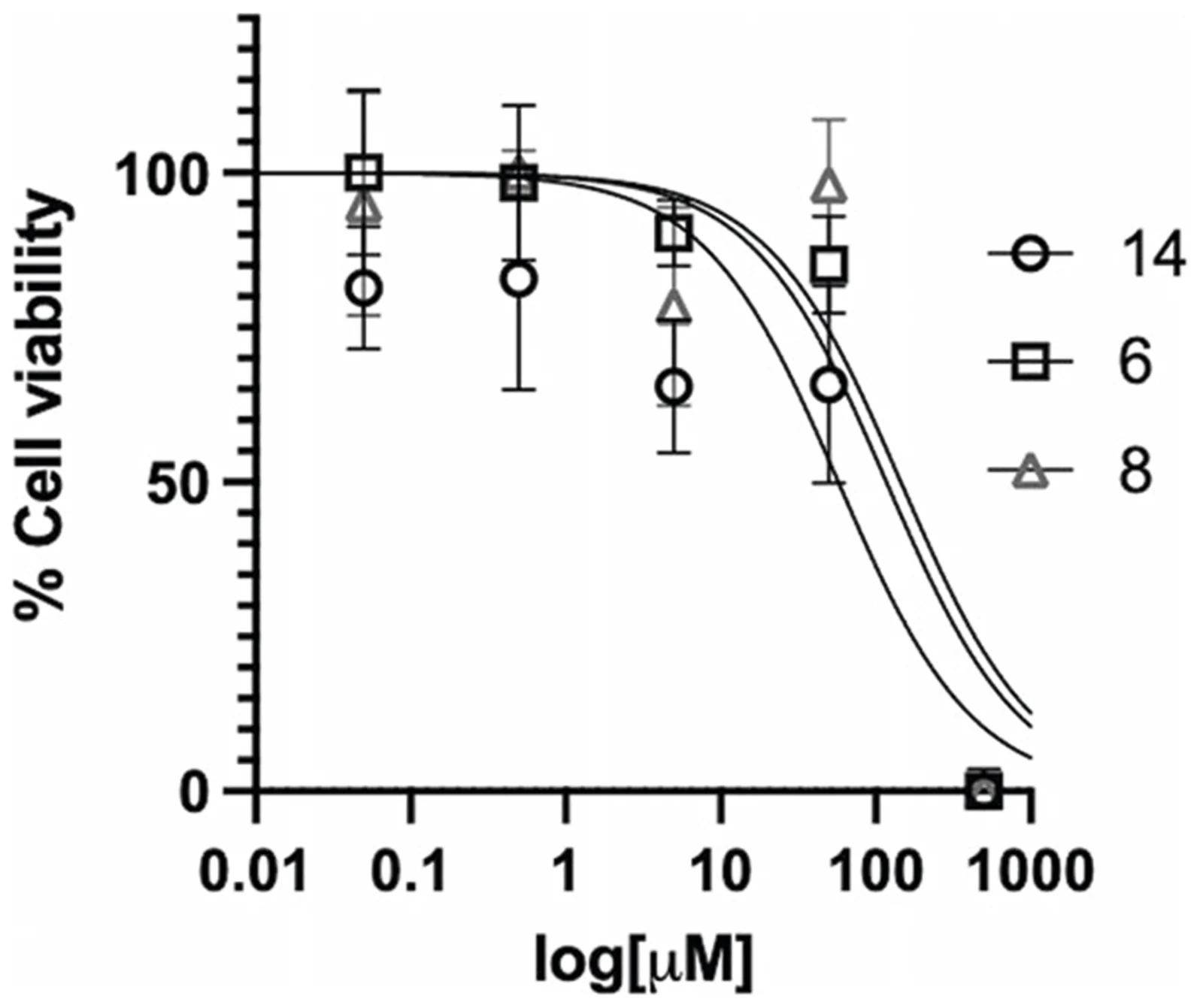
Cytotoxicity of compounds 6, 8, and 14 against RAW264.7 murine macrophages. Cells were treated for 24 hours and cell viability was determined using Cell-Titer Glo assay. Each data point is the result of three independent determinations ± SEM

**Fig. 7:**
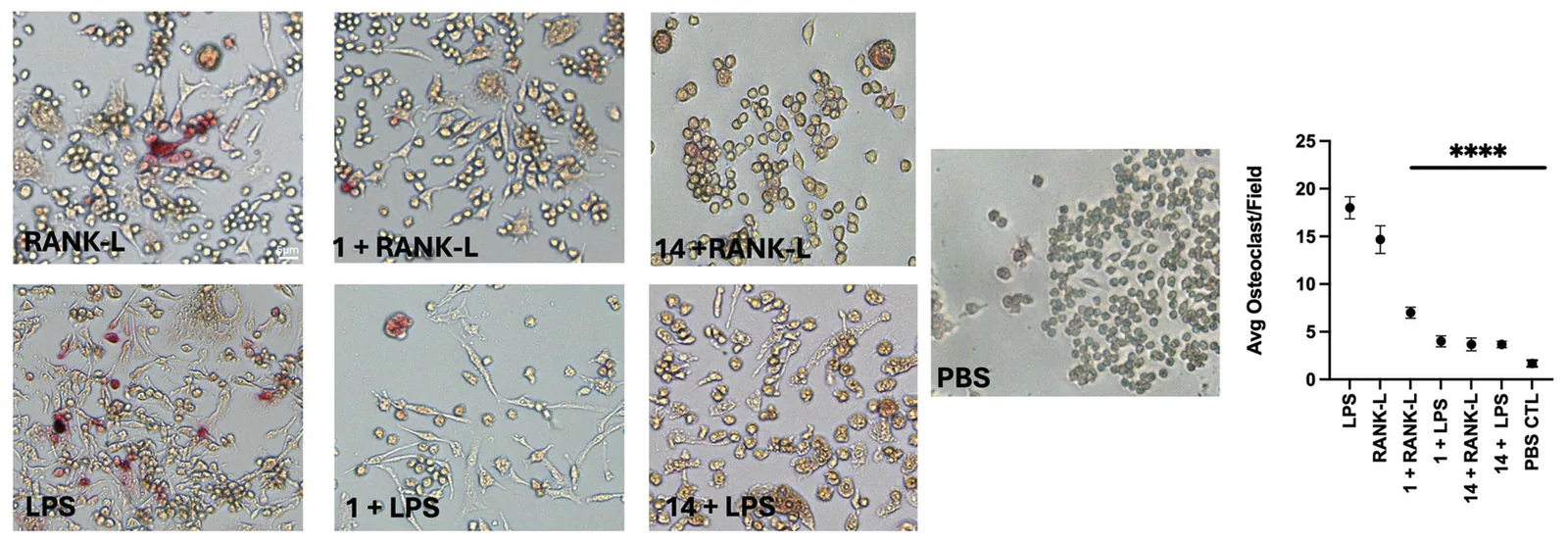
KDM4B inhibition prevents osteoclast formation induced by either Aa-LPS or RANK-L. Cells were differentiated into osteoclasts using RANK-L for 2 days. On the third day, cells were rinsed and pretreated for 1 hour with **1, 14**, or DMSO vehicle control followed by supplementation with RANK-L or Aa LPS. After 72 hours, cells were fixed and stained for Tartrate-Resistant acid phosphatase. 3 representative images were taken of each well, and TRAP+ multinucleated cells were counted in each field. Images are zoomed in representative fields of designated well. Data is presented as the mean number of osteoclasts in each field SEM. Statistical significance was determine using a paired Friedman test with multiple comparisons at an a = 0.05. *p < 0.01 *n* = 3fields per well, 3 wells per group

**Scheme 1:**
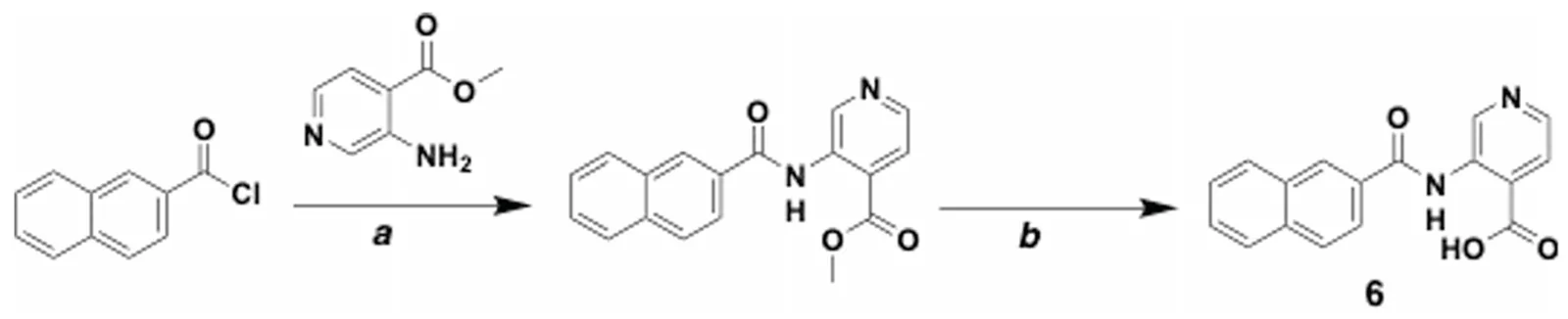
Reagents and Conditions: **a** Pyridine, DCM, rt, 12 h. **b** 1M LiOH, 3:1 MeOH H_2_O, rt, 8 h

**Scheme 2:**
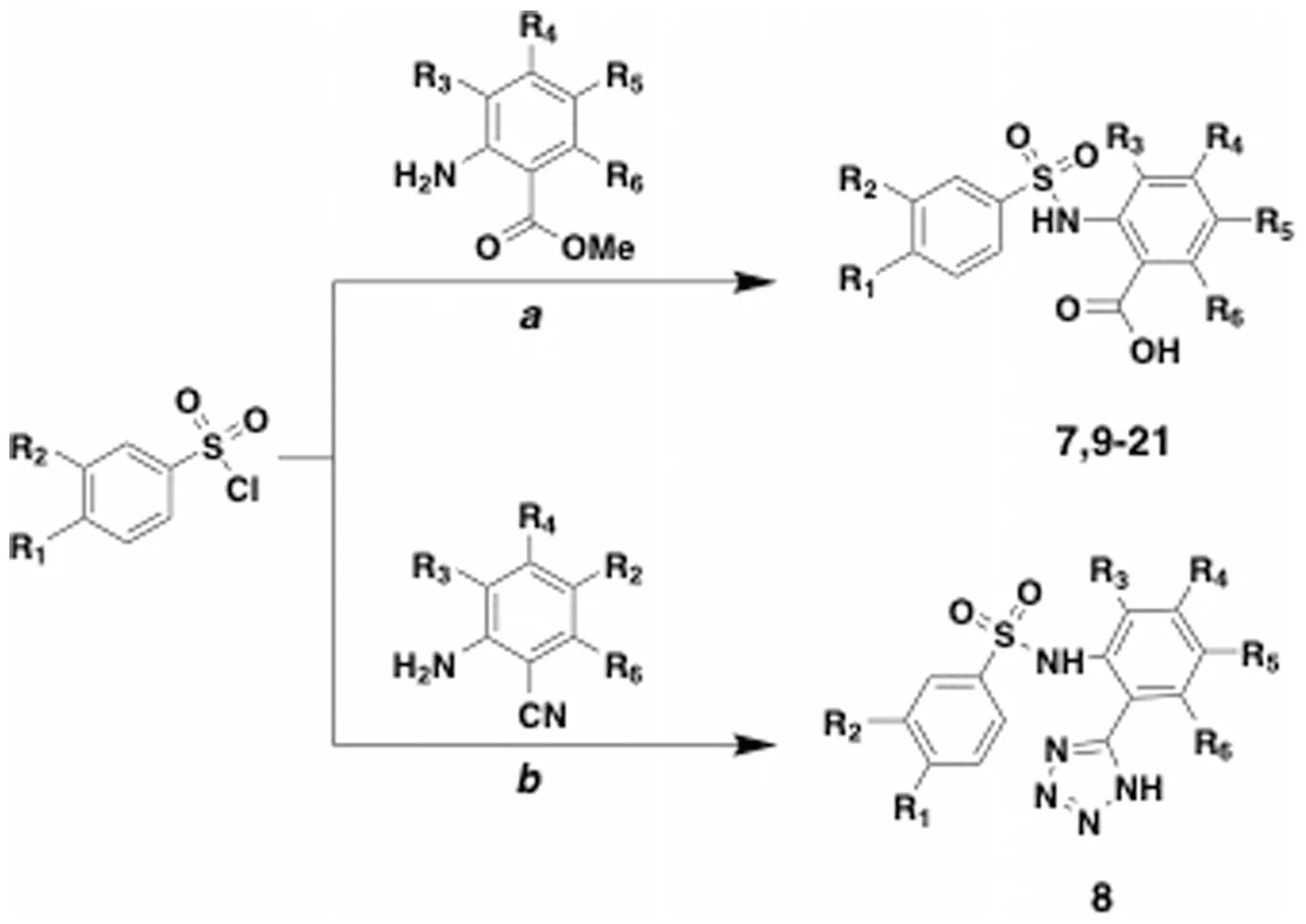
Reagents and Conditions: **a** 1. Pyridine, DCM, rt, 12 h. 2. 1 M LiOH, 1:1 THF:H_2_O, rt, 8 h. **b** 1. pyridine, DCM, rt, 12h ; (2) NaN_3_, CuSO_4_ · H_2_O, DMSO, 150 C, 1 h

**Table 1:** Table of KDM4B inhibitors and their respective IC_50_ values

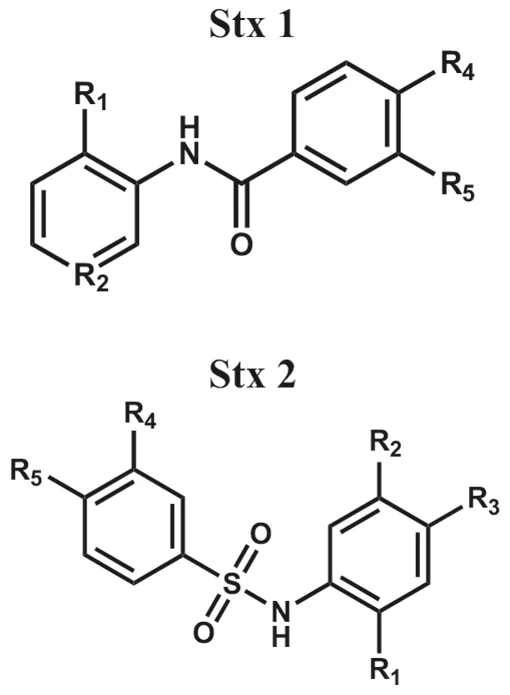							
Cmpd	Stx	R1	R2	R3	R4	R5	IC_50_ ± SEM (μM)^a^
6	1	COOH	N	H	CH = CHCH = CH		0.4 ± 0.2
7	2	COOH	F	F	H	4-MePh	0.6 ± 0.4
8	2	TTZ^b^	H	F	H	4-MePh	0.4 ± 0.2
9	2	COOH	H	CF3	H	4-MePh	1.0 ± 0.2
10	2	COOH	H	Br	H	4-MePh	0.38 ± 0.06
11	2	COOH	H	CI	H	4-MePh	ND
12	2	COOH	H	F	H	3-MePh	0.7 ± 0.5
13	2	COOH	H	F	H	4-OMe	ND
14	2	COOH	H	F	H	4-ClPh	0.17 ± 0.03
15	2	COOH	H	F	H	C6H11	ND
16	2	COOH	H	F	CH = CHCH = CH		ND
17	2	COOH	H	F	H		ND
18	2	COOH	H	F	H	4-FPh	2.3 ± 0.4
19	2	COOH	H	F	CH2CH2CH2CH2		1.0 ± 0.2
20	2	COOH	H	F	0CH2CH20		0.43 ± 0.07
21	2	COOH	H	Me	H	4-ClPh	0.36 ± 0.04

a IC_50_ values were determine for compounds exhibiting ≥50% inhibition at 20 μM

b TTZ represents tetrazole
