# Supplementary material for: Synthesis, characterization, and evaluation of KDM4B inhibitors to attenuate inflammatory host immune response in periodontitis
Source: Med Chem Res. Author manuscript; Available in PMC 2026 Oct 16. (PMC13674686; doi:10.1007/s00044-024-03362-5)
Supplement: Supplementary file 1 [file NIHMS2190912-supplement-Supplemental.docx]

**Synthesis, characterization, and evaluation of KDM4B inhibitors to attenuate inflammatory host immune response in periodontitis**

**Kathleen Garrabrant^a^, Amelia Furbish^a^, Jonathan Turner^a^, Ivett P. Gomez^a^, Catherine Mills^a^, Abhiram Maddi^b^, Yuri Peterson^a^**

*^a^ Department of Drug Discovery and Biomedical Sciences, Medical University of South Carolina; 70 President St, Charleston, SC, 29425, USA*

*^b^Department of Advance Specialty Sciences, Medical University of South Carolina, 173 Ashley Ave, Charleston, SC 29425, USA*

**Supplemental Information**

**Supplemental Figure 1: Enzyme Kinetics with recombinant KDM4B and compound** Panel A: Enzyme kinetics for human recombinant KDM4B and peptide substrate at 0.0, 0.125, and 0.5uM **14**. Panel B: Lineweaver Burk plot for the inhibition of KDM4B by **14** and 0.0, 0.125, and 0.5uM indicating uncompetitive inhibition kinetics. Each data point is the result of three independent determinations ± SEM. Collectively, these data suggest **14** exhibits mixed-uncompetitive inhibition against KDM4B enzyme.

**Supplemental Figure 2: IC50 confirmation with secondary Succinate-Glo™️ assay**

Panel A: IC50 with control compound ML324 (**1**) Panel B: IC50 with compound **14** Each data point is the result of three independent determinations ± SEM.

**99**

**Supplemental Figure 3: KDM4B inhibition prevents osteoclast formation induced by either Aa-LPS or RANK-L**. Cells were differentiated into osteoclasts using RANK-L for 2 days. On the third day, cells were rinsed and pre-treated for 1h with **1**, **14**, or DMSO vehicle control followed by supplementation with RANK-L or Aa LPS. After 72hours, cells were fixed and stained for Tartrate Resistant acid phosphatase. 3 representative images were taken of each well, and TRAP+ multinucleated cells were counted in each field. Data is presented as the mean number of osteoclasts in each field SEM. Statistical significance was determine using a paired Friedman test with multiple comparisons at an a=0.05. *p<0.01 n=3fields per well, 3 wells per group, and significance is compared to LPS control.

**Compound 6**

**Compound 7**

**Compound 8**

**Compound 9**

**Compound 10**

**Compound 11**

**Compound 12**

**Compound 13**

**Compound 14**

**Compound 15**

**Compound 16**

**Compound 17**

**Compound 18**

**Compound 19**

**Compound 20**

**Compound 21**
